# The tumour is not enough or is it? Problems and new concepts in the surgery of cerebral metastases

**DOI:** 10.3332/ecancer.2013.306

**Published:** 2013-04-18

**Authors:** Marcel A Kamp, Maxine Dibué, Antonio Santacroce, Samis MA Zella, Lena Niemann, Hans-Jakob Steiger, Marion Rapp, Michael Sabel

**Affiliations:** 1 Department for Neurosurgery, Medical Faculty, Heinrich Heine University, Moorenstraße 5, 40225 Düsseldorf, Germany; 2 Institute for Neurophysiology, University of Cologne, Germany; 3 Center of Molecular Medicine, Cologne, Germany; * Contributed equally

**Keywords:** cerebral metastases, surgery, supramarginal resection, local recurrence, fluorescence

## Abstract

Cerebral metastases are the most frequent cerebral tumours. Surgery of cerebral metastases plays an indispensible role in a multimodal therapy concept. Conventional white-light, microscopy assisted microsurgical and circumferential stripping of cerebral metastases is neurosurgical standard therapy, but is associated with an extraordinarily high recurrence rate of more than 50% without subsequent whole-brain radiotherapy. Therefore, neurosurgical standard therapy fails to achieve local tumour control in many patients. The present conceptual paper focuses on this issue and discusses the possible causes of the high recurrence rates such as intraoperative dissemination of tumour cells or the lack of sharp delimitation of metastases from the surrounding brain tissue resulting in incomplete resections. Adjuvant whole-brain radiotherapy reduces the risk of local and distant recurrences, but is associated with a well-documented impairment of neurocognitive function. New surgical strategies, such as supramarginal or fluorescence-guided resection, address the possibility of infiltrating tumour parts to achieve more complete resection of cerebral metastases. Supramarginal resection was shown to significantly reduce the risk of a local recurrence and prolongs two-year survival rates. Furthermore, radiosurgery in combination with surgery represents a promising approach.

## Introduction

The incidence of brain metastases appears to be rising, probably due to the improvements in systemic therapy leading to longer survival, an aging patient population, and the now widespread use of magnetic resonance imaging (MRI) [1]. Cerebral metastases are the most common intracranial neoplasm in adults, outnumbering primary brain tumours by a ratio of 10:1 [2] and affecting up to 170,000 patients each year in the USA alone. Therefore, cerebral metastases cause significant morbidity and mortality [1].

Surgery plays an indispensable role in the treatment of cerebral metastases, and the benefit of surgery has been documented in numerous studies. However, standard surgical treatment of cerebral metastases is often insufficient in achieving local tumour control, becoming obvious in the high local recurrence rate of surgically resected cerebral metastases without subsequent radiation therapy, which was estimated to be about 50% in some studies [3, 4]. Therefore, evaluation of available and future surgical approaches is of great importance. The present work discusses old and new approaches in the surgery of cerebral metastases.

## Impact of surgical therapy

Today, in the treatment of cerebral metastases, surgery in combination with subsequent whole-brain radiation therapy (WBRT) is standard protocol. Two hallmark randomised studies from the early 1990s showed the benefit of surgery with subsequent WBRT compared with WBRT alone [5, 6]. A later randomised trial found no improvement in the outcome of patients with a single brain metastasis that underwent surgery in addition to WBRT, albeit these patients suffered from a systemic progression more frequently than from neurological recurrence [7]. Furthermore, several retrospective analyses documented the benefit of surgery in addition to WBRT compared with WBRT alone [8, 9]. These studies document the indispensible role of surgery in the treatment of cerebral metastases.

## Standard surgical therapy

In order to achieve complete tumour resection and to prevent postoperative neurological deficits, conventional white-light microscopy-assisted microsurgical and circumferential stripping of cerebral metastasis from the surrounding brain parenchyma is employed and has become neurosurgical standard therapy. Furthermore, evidence suggests that an *en bloc *resection may be beneficial compared with piecemeal resection for supra- and infra-tentorial situated cerebral metastases. In addition to the type of resection, preoperative tumour volume significantly influences the incidence of local recurrence [10–12]. However, although complete *en bloc *resection should be aspired to, is not possible in all patients when metastases are localised in eloquent-brain areas and are adherent or infiltrate adjacent brain tissue.

Standard procedure of metastasis surgery includes several modern comforts of neurosurgery including: intraoperative use of navigation systems that help in planning the craniotomy; localising the tumour and eloquent structures; and minimising searches for the metastasis within the brain parenchyma, therefore reducing perioperative morbidity, thus improving surgical outcome [13, 14]. Intraoperative use of ultrasound imaging also assists intraoperative tumour localisation and may offer greater insight into the delineation of metastases from the adjacent brain tissue, thus improving the extent of surgical resection [15–17].

## Insufficiency of surgical resection alone

As discussed previously, complete surgical tumour resection with low morbidity and mortality is the goal in surgery of cerebral malignancies. Complete surgical resection is important to avoid local recurrences at the original site, as local relapses are most likely caused by incomplete tumour destruction or by intraoperative dissemination of tumour cells, for example, by a piecemeal resection. Therefore, local recurrences of cerebral metastases must be valued as a consequence of failure of the initial therapy.

Local recurrence of cerebral metastases after surgical resection without a subsequent WBRT is frighteningly frequent, and occurred in the range of 70% in older studies [18]. More recently, two authors reported the relapse rate of patients that underwent gross-total resection without subsequent WBRT to be between 50 and 60% [4,19]. Interestingly, this high recurrence rate occurred although tumour-cell free margins were histopathologically confirmed in the study of *Yoo et al *[4], and complete surgical resection was proven by MRI in nearly 75% of patients in the study of Kocher *et al *[19]. From this data, showing recurrence rates around 50% despite complete surgical resection, one must conclude that standard surgical resection alone is insufficient in achieving long-lasting tumour control.

## Infiltration of cerebral metastases

Despite the urgency of the question, the reasons for which cerebral metastases locally relapse at such a high rate despite complete surgical resection has yet to be fully understood. In addition to intraoperative dissemination of tumour cells by a piecemeal resection technique, the growth pattern of cerebral metastases may be the cause of their local recurrence. Cerebral metastases are widely believed to be sharply delimitated from the surrounding brain tissue and thus easily dissected from adjacent brain tissue, however, newer data suggest otherwise. Invasive growth patterns of cerebral metastases were reported from more than 50% of patients, who underwent brain autopsies [3]. Specially small-cell lung cancer and melanoma frequently invade adjacent brain tissue often by more than 1 mm in depth [3, 20]. Highly diffuse pseudogliomatous invasion patterns have also been observed in another series of intracerebral anaplastic small cell and adenocarcinomas [21]. Furthermore, less than 40% of tumours in our recent series displayed sharp delimitation from the adjacent brain parenchyma, whereas over 60% of tumours demonstrated irregular tumour–brain interfaces with tongue-like extensions of tumour tissue and small perivascular islets of tumour cells in the adjacent brain parenchyma [22]. Taken together, irregular tumour–brain interfaces appear to be very common in patients with cerebral metastasis and may account for high recurrence rates.

## Impact of postoperative radiotherapy

Infiltrating tumour parts and intraoperatively disseminated tumour cells are potentially destroyed by a postoperative radiotherapy, which has shown to be beneficial in several studies. Surgery plus WBRT therapy is superior to surgery as addressed in one prospective randomised trial [18] and different retrospective studies [23–25]. However, a routine postoperative resection control of surgery documenting the sufficiency of surgery was not performed. Recently, the results of the EORTC 22952-26001 trial analysing the impact of WBRT following surgery or radiosurgery have been published. In the surgery arm, WBRT reduced two-year relapse rates at the initial site (59% versus 27%, *p *= 0.001) and at new sites (42% versus 23%; *p *= 0.008) [19]. However, WBRT failed to affect overall survival (*p *= 0.89) and duration of functional independence [19]. About three-fourth of patients in the surgery arm of the EORTC 22952-26001 study received a postoperative MRI or computed tomography (CT) [19].

Furthermore, the WBRT concept came from a view that there are usually multiple metastases in part not visible by conventional imaging. These metastases will develop at new sites and might be treated by WBRT. In fact, WBRT halved the risk of new metastases at new sites in the EORTC 22952-26001 study, which was 43% and 48% for the surgery and radiosurgery arm, respectively [19]. However, although evidence is rather poor, radiosurgery without subsequent WBRT seems to be beneficial compared with WBRT alone, as patients showed a significantly longer median survival time in a prospective three-armed trial [26]. Therefore, these radiosurgery data imply that treatment of conventional visible metastases impacts survival time to a greater extent than treatment of non-visible potential micrometastases.

WBRT also continues to be problematic, bearing severe side effects such as radiation-induced neurocognitive impairment with an acute neurocognitive impairment and a delayed irreversible decline of neurocognitive function [27]. Neurocognitive impairment due to WBRT is well-documented [27]. An alternative therapy concept to surgery and subsequent WBRT might be radiosurgery of cerebral metastases alone, or surgery and subsequent radiosurgery of the resection cavity. In fact, one study found radiosurgery alone to achieve tumour control rates which were as good as those achieved by surgery and subsequent WBRT [28]. Furthermore, surgery and subsequent radiosurgery of the tumour bed seem to be a promising approach and are alternative to WBRT following surgery. Some publications and abstracts suggest that there might be a benefit [29]. However, evidence for this approach is lacking without a single published prospective series. Perhaps the ongoing Intergroup trial N107C comparing WBRT with surgical bed radiosurgery might give new insights. A further concept might be surgery and subsequent conformal radiotherapy instead of WBRT. However, this concept has no evidence, and has to be evaluated in prospective studies.

Besides, the use of monoclonal antibody is a hallmark approach, improving therapy and prognosis of patients suffering from malignant diseases. Nevertheless, development and evaluation of new invasive and non-invasive treatment regimes for cerebral metastases are urgently required and may ultimately reduce the necessity of WBRT, which so often comes in hand with neurocognitive deficits.

## Fluorescence-guided resection

5-Aminolevulinic acid (5-ALA)-derived fluorescence-guided resection was introduced in surgery of malignant gliomas about a decade ago. This technique was shown to enable more complete resections of malignant gliomas and improve local tumour control and six month progression-free survival compared with conventional white-light resection [30–32]. 5-ALA fluorescence-guided resection has since become a neurosurgical standard therapy in many neuro-oncologic centres. However, 5-ALA-induced fluorescence (5-AIF) occurs also in various tumours outside the central nervous system such as in lung, prostate, colorectal, and bladder carcinomas [33–39]. It is part of different treatment concepts established for these malignancies. Therefore, 5-AIF may visualise cerebral metastases as well as infiltrating tumour parts and subsequently may enable more complete resection of cerebral metastases.

Initially, Utsuki *et al*. analysed 5-AIF in a small series of 11 metastatic brain tumours and found 5-AIF in 9/11 patients (82%) [41]. This observation is supported by a later study, which found 5-AIF in 32/52 (62%) intracerebral metastases [22]. However, in the latter study, 5-AIF was neither associated with the histological type nor with the primary tumour itself, and fluorescence in positive metastases was heterogeneously distributed throughout the tumour tissue [22].

Both studies also analysed fluorescence of the tumour cavity after white-light assisted, macroscopically complete tumour resection, and evaluated the impact of 5-AIF in the detection of possible infiltrating tumour parts. Utsuki and colleagues observed 5-AIF not only in peritumoural but also in tumour-free oedematous tissue and assumed an unspecific protoporphyrin IX leakage [41]. In the second study, residual fluorescence of the resection cavity occurred in 60% of patients, and residual metastatic tumour tissue was histopathologically proven in the white-light, normal-appearing but fluorescent adjacent brain tissue in one-third of patients [22]. Interestingly, the resection cavity can exhibit a strong 5-AIF even in metastases with little or no 5-AIF, supporting the theory of an unspecific leakage of the peritumoural oedematous tissue [22].

As the majority of intracerebral metastases exhibit 5-AIF fluorescence, this method could improve visualisation of the surgical target. The intense fluorescence of the peritumoural adjacent brain tissue, even of metastases with little or no 5-AIF, may prove useful in locating tumour tissue. However, cerebral metastases themselves display regionally heterogeneous and unpredictable 5-ALA-induced fluorescence patterns, which do not correlate with histopathological findings. Also, reliable identification of infiltrating tumour parts appears to be problematic, as fluorescence of the adjacent brain tissue correlated with histopathological verification of tumour tissue in only one-third of patients. Therefore, 5-AIF of the adjacent peritumoural brain tissue should be interpreted with great caution.

## Supramarginal resection of cerebral metastases

One key study analysing new approaches in the surgery of cerebral metastases was performed by Yoo *et al. *[4]. Supramarginal resection was performed in 43 patients with cerebral metastases situated in non-eloquent brain areas by extension of the resection to a depth of about 5-mm after complete microsurgical resection. Margins of the resection cavity were proven to be tumour cell-free by frozen section. Results of these patients were compared with another group of 51 patients with eloquent-situated cerebral metastases that underwent conventional gross-total resection (*p *= 0.003; two-year recurrence rates were 29.1% and 63.2% for the supramarginal resection and control group, respectively) [4]. Supramarginal resection of cerebral metastases significantly reduced the risk of a local recurrence in a multivariate analysis. Furthermore, two-year survival rates were significantly improved in the supramarginal resection group (*p *= 0.0001) [4]. Furthermore, awake surgery and adequate electrophysiological monitoring enable supramarginal resection in eloquent localised cerebral metastases at a low risk of newly occurring postoperative neurological deficits [40].

## Conclusions

Although surgery has proven indispensible in the treatment of cerebral metastases, new surgical techniques in combination with noninvasive methods are necessary in enhancing tumour control. Conventional white-light microscopy-assisted microsurgical and circumferential stripping of cerebral metastasis from the surrounding brain parenchyma remains standard as fluorescence-guided resection does not appear to be reliable in identifying infiltrating tumour parts. Furthermore, *en bloc *resection may be beneficial compared with piecemeal resection. Nevertheless, local recurrence of cerebral metastases after surgical resection without subsequent WBRT is frighteningly frequent (more than 50%) after complete surgical resection. Explanations for these high recurrence rates may be an intraoperative dissemination of tumour cells, or the lack of sharp delimitation of the metastasis from the surrounding brain tissue resulting in incomplete resection. WBRT reduces the risk of a local recurrence but comes at a very high cost. Supramarginal resection of cerebral metastases (which significantly reduces risk of a local recurrence and prolongs two-year survival rates), and radiosurgery in combination with surgery represent promising approaches and may prove essential in improving treatment of cerebral metastases.
